# WiFi-Based Human Identification with Machine Learning: A Comprehensive Survey

**DOI:** 10.3390/s24196413

**Published:** 2024-10-03

**Authors:** Manal Mosharaf, Jae B. Kwak, Wooyeol Choi

**Affiliations:** 1 Department of Computer Engineering, Chosun University, Gwangju 61452, Republic of Korea; manal@chosun.ac.kr; 2 Department of Mechanical Engineering, Chosun University, Gwangju 61452, Republic of Korea; jaekwak@chosun.ac.kr

**Keywords:** WiFi, human sensing, human identification, machine learning, deep learning

## Abstract

In the modern world of human–computer interaction, notable advancements in human identification have been achieved across fields like healthcare, academia, security, etc. Despite these advancements, challenges remain, particularly in scenarios with poor lighting, occlusion, or non-line-of-sight. To overcome these limitations, the utilization of radio frequency (RF) wireless signals, particularly wireless fidelity (WiFi), has been considered an innovative solution in recent research studies. By analyzing WiFi signal fluctuations caused by human presence, researchers have developed machine learning (ML) models that significantly improve identification accuracy. This paper conducts a comprehensive survey of recent advances and practical implementations of WiFi-based human identification. Furthermore, it covers the ML models used for human identification, system overviews, and detailed WiFi-based human identification methods. It also includes system evaluation, discussion, and future trends related to human identification. Finally, we conclude by examining the limitations of the research and discussing how researchers can shift their attention toward shaping the future trajectory of human identification through wireless signals.

## 1. Introduction

In recent years, detection technologies have advanced significantly across various fields, including object detection, environmental monitoring [1], remote sensing images (RSIs) [2,3], wildlife monitoring using acoustic sensors [4] and cameras, and human sensing. Human behavior or activity recognition has drawn a lot of interest because of the recent advancement of information technology and the rise in safety and security concerns of individuals in domains such as the healthcare system [5]. Virtual reality, motion analysis, smart homes, and other applications of human behavior recognition are frequently used and have significant research implications [6,7,8]. Human identification (HID) is at the center of these applications and serves as a fundamental requirement for various applications that prioritize comfort, security, and privacy. HID is the fundamental technology for applications such as smart buildings [9], intruder detection [10], and area access control [11]. Therefore, accurate identification of individuals is critical for implementing and improving these types of technologies and systems. For precise person identification, various unique biological characteristics or behaviors have been thoroughly studied and utilized by researchers. These include fingerprints [11], iris patterns [12], voiceprints [13], vital signs [14,15], and gait patterns [16].

### 1.1. Approaches to Human Identification

HID approaches are classified as device-based or device-free, depending on whether the subject wears or carries the sensor. Device-based sensing approaches involve vision-based systems, wearable devices, and biometric information-based systems. In vision-based systems, computer vision has received considerable attention, utilizing cameras for HID [17,18]. Gait analysis for HID requires analyzing both general gait characteristics and complex distinctive walking subtleties. The two main techniques that are often used in gait analysis are the silhouette-based method, which concentrates on overall body shapes during mobility, and the model-based approach, which builds intricate models of human body motion during walking. Both approaches have shown notable advancements in the area of HID using gait analysis in computer vision (CV) [19]. Wearable device-based systems utilize smartphones or smart tags for HID [20,21], while biometric information-based systems employ fingerprints [22,23] and retinal scans [24,25]. Device-based approaches are generally accurate and achieve high precision, although they can encounter challenges in real-world situations. For instance, the CV approach has some drawbacks, such as privacy issues, difficulty in low light, inability to pass through walls, and challenges identifying people in obstructed locations [26]. In wearable device-based systems, it is challenging to detect intruders or unknown users, and patients or the elderly must carry the device continuously, presenting practical difficulties. Biometric information-based systems, while capable of achieving high accuracy, involve high costs and the sensitive collection of data through fingerprint and retinal scan devices.

Recent developments in wireless technologies have been seen as a solution to these problems [27]. Contrarily, device-free techniques such as radio frequency (RF) signals address these restrictions, as they are capable of protecting privacy, can pass through barriers like walls, and work well in dim lighting. In this technique, the system tries to understand human existence and identification using signals without any camera or wearable device; rather, it uses human behavior, gait, or gesture patterns by analyzing the changes and reflections in the RF signals [28].

### 1.2. Rf-Based Approaches

Researchers have proposed wireless technology solutions, particularly utilizing RF technology such as wireless fidelity (WiFi) sensing and radar imaging, to improve HID methods. Employing RF sensing, these technologies can detect and reflect signals from a walking human, resulting in distinctive signal variations caused by unique human biometrics such as body shape, size, and movement patterns. By analyzing these variations, RF signals can provide detailed information about an individual’s gait, posture, and gestures. This detailed analysis of RF signal variations suggests significant potential for innovative identification applications, offering a privacy-preserving, non-intrusive alternative to device-based methods. Current RF-based sensing systems utilize either readily available WiFi devices or specialized RF equipment [29]. In the subsequent section, we will delve into the details of how studies have utilized numerous features such as micro-Doppler structure, gait analysis, gesture recognition, radio biometrics, and respiratory patterns for HID.

#### 1.2.1. Radar Imaging

In the realm of non-contact and non-line-of-sight perception, radar imaging stands out as a powerful technique for HID [30]. Comparable to data collected by sensors, a multitude of wireless signals can be harnessed for this purpose [31]. For example, radar sensing often employs dedicated devices to discern the target’s location, shape, and motion characteristics [32,33,34]. Radar technology emits radio waves that travel through the environment. When these waves encounter objects, they bounce back as echoes. By analyzing the time it takes for these echoes to return and their characteristics, such as frequency shifts due to motion (Doppler effect) and amplitude changes, radar systems can determine the distance, speed, and even certain properties of the objects. This information is then processed to create a radar image or extract meaningful data, enabling applications like gesture recognition, object detection, and presence sensing [35].

Within the realm of radar imaging, two fundamental radar system classifications emerge: frequency-modulated continuous wave (FMCW) radar and ultra-wideband (UWB) radar. These two categories possess distinct strengths. FMCW radar is cost-effective and advantageous in specific domains, making it a popular choice [36]. Both FMCW and UWB radar systems utilize the micro-Doppler effect. This effect involves frequency changes caused by micro-motions, such as vibrations or rotations, in addition to the main motion of the target. These micro-motions cause additional frequency shifts around the main Doppler signal of the target. By analyzing these shifts, radar systems can gather detailed information about the target’s specific movements and behaviors. This analysis of micro-Doppler patterns improves the radar’s ability to identify and classify targets [37]. By leveraging these micro-Doppler structures, both FMCW and UWB radar systems play significant roles in advancing the identification of humans through the nuanced analysis of these intricate motion patterns.

**Figure 1:**
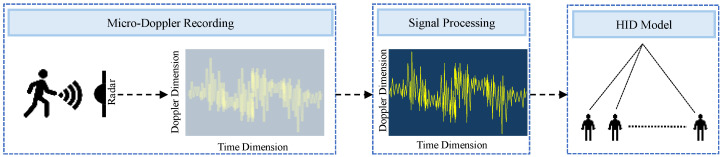
Micro-Doppler analysis with FMCW radar [38,39].

Micro-Doppler analysis with FMCW radar: Micro-Doppler analysis with FMCW radar investigates Doppler frequency modulations caused by micro-motions using FMCW radar. In FMCW radar, a continuous wave is modulated in frequency over time, sending out a wave with a linearly increasing or decreasing frequency. The radar extracts Doppler information by analyzing the frequency difference between the transmitted and received signals. This approach captures detailed features such as frequency shifts and gait cycle information, providing insights into intricate movement patterns during activities like walking. The FMCW radar effectively detects unique motion patterns from different body parts, including the torso, arms, and legs. By analyzing these micro-Doppler features, the radar distinguishes individuals based on their distinct gait patterns, which is valuable for open-set HID [38]. This capability highlights FMCW radar’s versatility in human recognition and person identification. For instance, one study [28] emphasizes the FMCW radar’s capability to analyze micro-Doppler signatures, revealing intricate movement patterns during walking, crucial for human recognition and movement classification. Another research effort [39], employing a 77 GHz FMCW radar, focuses on Doppler frequency shifts (DFSs), sidebands, and their patterns in micro-Doppler analysis. Figure 1 illustrates the working principle of FMCW radar.

Micro-Doppler analysis with UWB radar: Micro-Doppler analysis with UWB radar leverages UWB radar to study Doppler frequency changes caused by small movements. UWB radar emits extremely short-duration pulses, typically in the nanosecond range, and has a broad bandwidth. This combination allows UWB radar to achieve high resolution in both range and velocity, making it particularly effective for detailed analysis of micro-Doppler signatures. The wide bandwidth and short pulses enable UWB radar to better distinguish closely spaced objects and capture finer details in the Doppler domain. This precision is crucial for accurately capturing and analyzing micro-Doppler spectrograms generated by various human movements. For instance, a study by Yang et al. [40] employs a UWB radar operating at 4.3 GHz with a pulse repetition frequency (PRF) of 368 Hz. This monostatic radar system uses the same antenna for both transmitting and receiving signals, allowing it to capture micro-Doppler spectrograms reflecting different segments of the human body during various motions.

#### 1.2.2. WiFi Sensing

WiFi sensing is an emerging technology that utilizes WiFi signals to interpret and understand the surrounding environment. It supports various applications, including indoor localization, human activity recognition (HAR) [41], environmental monitoring, pose estimation [42], and HID. Despite the challenges in interpreting WiFi signals and extracting meaningful insights, WiFi sensing presents several advantages over radar-based methods. It leverages existing infrastructure, simplifying deployment and reducing costs. The higher frequency of WiFi signals improves resolution, enhancing the accuracy of movement tracking. Unlike radar technology, which struggles with wall penetration, WiFi sensing provides comprehensive coverage without blind spots. Additionally, the affordability and widespread availability of WiFi make it an efficient choice for detecting a presence within its range.

Early WiFi-based systems relied on received signal strength (RSS) for basic sensing [43,44]. However, RSS can be inconsistent and subject to random variations. Recent advancements have shifted focus to channel state information (CSI), which offers more stable and precise data. By utilizing frequency diversity [45] and the detailed signal information from CSI, researchers have significantly advanced HID techniques [46,47,48,49,50,51,52,53]. This survey offers a comprehensive review of HID using WiFi sensing technologies, particularly CSI. It examines how CSI data are used to analyze various aspects of human behavior, including gait, gestures, radio biometrics, and respiration rate.

### 1.3. Machine Learning for WiFi Sensing

Machine learning (ML) has become pivotal in advancing WiFi sensing technologies, enabling more sophisticated and accurate interpretations of WiFi signals [54]. By leveraging ML algorithms, researchers can extract meaningful patterns and features from CSI data. This enhances the effectiveness of WiFi-based HID [55]. These algorithms are categorized into two main approaches: conventional statistical methods and deep learning (DL) models. Conventional methods, including support vector machines (SVMs) [50] and Gaussian mixture models (GMMs) [53], utilize mathematical techniques to classify and predict based on extracted features. In contrast, deep learning methods, such as convolutional neural networks (CNNs) [49], recurrent neural networks (RNNs) [56], and hybrid models [57], excel in handling complex and high-dimensional data. Recent advancements in artificial intelligence (AI) have further driven innovations in this space, allowing for more efficient data processing and enhanced feature extraction capabilities [58]. This drives innovation in areas ranging from user authentication to real-time environmental monitoring. In Section 3, we will discuss in detail WiFi CSI-based HID, covering the entire process from data collection (including gait, gesture, radio biometrics, and respiration rate) to the application of various ML models used for HID.

### 1.4. Contribution

Several surveys have focused on specific aspects of WiFi CSI sensing, each emphasizing different applications. For instance, the survey in [46] provides a broad overview of various applications, including human detection, motion detection, and respiratory monitoring. Ref. [59] conducted an extensive survey on CSI-based behavior recognition applications over six years, offering the most comprehensive temporal coverage. Another survey by [60] concentrates on human localization, detailing various methodologies and findings in this area. Similarly, the survey in [42] explores skeleton-based human pose recognition using CSI, presenting a focused analysis of this application. This survey is the first to comprehensively address WiFi CSI-based HID through ML. By focusing exclusively on this topic, it provides a thorough and distinctive analysis that sets it apart from previous surveys. Our main contributions can be summarized as follows.

- First comprehensive review: this survey is the first to focus exclusively on HID using WiFi CSI signals. It offers a systematic review of the entire identification process, from data collection to application, serving as a practical guide for implementing CSI technology in real-world scenarios.
- Focused analysis of CSI data for human identification: we provide a focused analysis of how channel state information (CSI) is utilized in human identification, emphasizing the processes involved in collecting CSI data through movements such as gait and gestures, as well as radio biometrics.
- Review of machine learning models: this survey reviews various ML models applied to CSI-based HID, detailing their methodologies, performance results, and effectiveness in identifying individuals.
- Discussion and future challenges: we discuss unique characteristics considered in different studies, such as human intruder detection and the use of bio-physiological signals (BVPs) alongside CSI. We also highlight future challenges, including the need for more robust models and improved integration of multi-modal data for enhanced accuracy and reliability.

**Figure 2:**
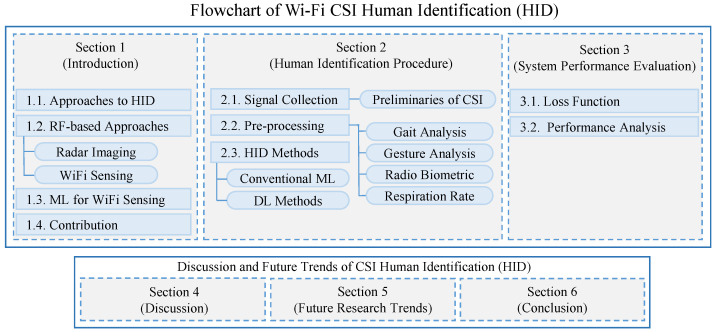
Taxonomy of this survey.

The structure of our survey is illustrated in Figure 2. The rest of the paper is organized as follows, where Section 2 provides a detailed examination of the HID procedure, divided into several key areas. Section 2.1 covers signal collection, including the preliminaries of CSI. Section 2.2 focuses on signal pre-processing, with subsections dedicated to analyzing gait, gestures, radio biometrics, and respiration rates using CSI. Section 2.3 reviews ML models for HID, encompassing both conventional statistical methods and advanced deep learning approaches. Section 3 evaluates system performance, while Section 4 and Section 5 discuss unique characteristics and future challenges, respectively.

## 2. Human Identification Procedure

**Figure 3:**
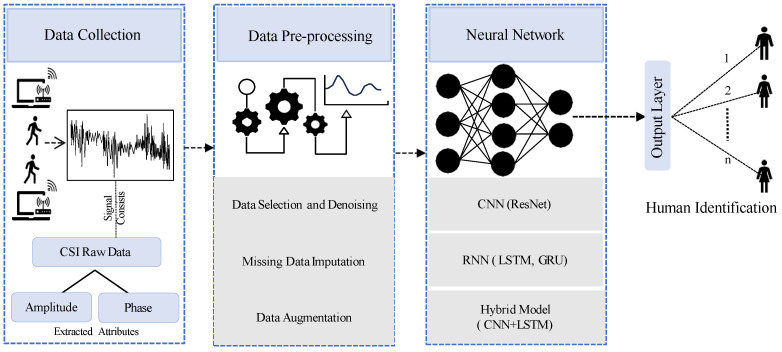
System overview of human identification.

To detect human presence using WiFi or laptop-based CSI data, researchers have developed a structured approach involving three main phases: data collection, pre-processing, and ML. Initially, CSI signals are systematically gathered to capture detailed information about the WiFi environment affected by human activities. Subsequently, the collected data undergo essential pre-processing steps, including the extraction of relevant CSI features, noise reduction, data segmentation, and sample selection, aimed at enhancing data quality and relevance. Once prepared, the processed data are analyzed using ML models. These models are tailored to effectively identify human identity based on the refined CSI data. Figure 3 in the literature illustrates a comprehensive system overview of the HID process. This survey section will delve into specific details of the CSI data types collected, the pre-processing techniques applied, and the diverse range of models employed, as documented in existing research on HID using WiFi technology.

### 2.1. Signal Collection

HID and human position information are mostly contained within CSI amplitude and phase. To obtain detailed insights into the signal collection process, we studied how WiFi CSI data are utilized in current research on HID. In the following section, we will discuss fundamental aspects of CSI, along with specific types of data collected such as gait analysis with CSI, gesture analysis with CSI, radio biometric analysis with CSI, and respiration rate analysis with CSI.

When it comes to WiFi technology, CSI is a fundamental and necessary component that provides crucial insights into the intricate details of wireless communication channels. It surpasses traditional measurements like RSSI [45]. It includes processes including scattering, attenuation, diffraction, fading, and reflection. These processes capture complex information regarding signal transmission. This level of detailed information provided by CSI is essential for optimizing WiFi networks and understanding the complexities of wireless environments. Raw CSI data are hard to work with, as they consist of a high level of noise ratio. However, by applying filtering or optimization processes, it is possible to calculate CSI using WiFi technology. This thorough understanding is enabled by multiple WiFi technologies such as orthogonal frequency division multiplexing (OFDM) and multiple-input multiple-output (MIMO).

OFDM modulation divides the WiFi spectrum into orthogonal carriers and subcarriers. By broadcasting data in parallel across several subcarriers, this division enables effective usage of the frequency spectrum that is accessible. MIMO technology increases data speed and spectrum efficiency by using multiple antennas on both the sending and receiving ends. It improves the reliability and capacity of wireless networks through spatial diversity and multipath propagation. Additionally, WiFi operates across multiple channels with frequencies around 2.4/5 GHz and bandwidths of 20/40/80/160 MHz. During propagation, these carriers change the frequency amplitude, phase, or power because of the Doppler effect. CSI is a measurement that represents these disparities between carriers and subcarriers. The signal received by the receiver can be expressed using the CSI as follows.

(1) $$y_{i}=H_{i}x_{i}+\eta_{i},$$

where, *i* denotes the specific subcarrier; $x_{i}\in R^{N_{T}}$ represents the transmitted signal, with $N_{T}$ being the number of transmitter antennas; $y_{i}\in R^{N_{R}}$ is the received signal, where $N_{R}$ is the number of receiver antennas; and $\eta_{i}$ is the noise vector, representing the noise in the received signal. $H_{i}$ denotes the CSI matrix of subcarrier *i*, which can be further detailed as a matrix of complex values.

(2) $$H_{i}=\left[\begin{array}{cccc} h_{11} & h_{12} & \ldots & h_{1N_{T}}\\ h_{21} & h_{22} & \ldots & h_{2N_{T}}\\ \vdots & \vdots & \ddots & \vdots \\ h_{N_{R}1} & h_{N_{R}2} & \ldots & h_{N_{R}N_{T}} \end{array}\right],$$

here, $h_{mn}=j\left|h_{mn}\right|e^{j∠h_{mn}}$ represents the complex value of $H_{i}$, where $|h_{mn}|$ is the amplitude and $∠h_{mn}$ is the phase of the CSI for the link between the *m*-th receiver antenna and the *n*-th transmitter antenna. For the human sensing application, where the human body affects the propagation environment [61], CSI in (2) captures the status of the environment. Specialized network interface cards (NICs) [62] are utilized to collect these CSI data. The Intel 5300 and Qualcomm Atheros series NICs are well-known solutions. The type of NIC and selected bandwidth affect the number of subcarriers available for CSI data collection. For instance, in a 20 MHz bandwidth scenario, Intel 5300 NICs offer 30 CSI subcarriers, whereas Qualcomm Atheros series NICs provide 56. Recent advancements have enhanced the capabilities of these NICs. For example, ref. [63] developed “Splicer”, which improves power delay profile resolution by combining CSI from multiple frequency bands, leading to better localization accuracy.

Additionally, several public CSI datasets have made important contributions to research in this field. Widar3.0 [64] has 258,000 hand gesture instances with advanced features such as DFSs. The WiAR [65] dataset features data from 10 volunteers performing 16 activities, achieving over 90% accuracy. The SignFi [66] deep learning algorithm recognizes 276 sign language gestures using CSI and demonstrates high accuracy in a variety of environments. Finally, eHealth [67] CSI supports remote patient monitoring by including diverse CSI data and participants’ phenotype information.

### 2.2. Signal Pre-Processing

In signal pre-processing, the raw CSI data measured by commodity WiFi devices cannot be used directly for HID due to environmental noise, unpredictable interference, and outliers. The signal pre-processing method plays a key role in extracting HID features, and typically involves CSI link selection, feature extraction, and denoising. The collected CSI data are gathered from various approaches, including human gait analysis, gesture analysis, radio biometrics, and even respiration rates. In the following section, we will discuss how researchers pre-process and extract parameters such as amplitude, phase, and the time-frequency domain from these approaches.

#### 2.2.1. Gait Analysis with CSI

**Figure 4:**
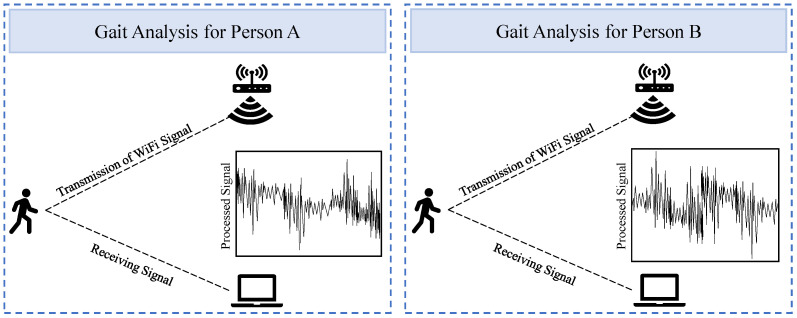
Operational scenario for Gate-ID. The WiFi CSI of two people’s gaits has unique patterns in the central area of the effective region [51].

Gait analysis using CSI involves using WiFi signal reflections from a moving human body to identify detailed walking patterns. The movement of the human body creates unique variations in CSI due to the multipath effect of wireless signals. As illustrated in Figure 4, the CSI signal is noticeably affected when a person walks between WiFi devices, and individuals exhibit different signal patterns. Recent studies on CSI-based gait analysis have demonstrated promising results. For example, ref. [16] investigated the time-frequency domain and extracted gait details by analyzing CSI variations. However, extracting information related to human identity from both the amplitude and phase of the signal is crucial.

Amplitude extracted from CSI cannot be directly used for identifying individuals based on gait patterns due to noise and signal variations. To address this, researchers employ various denoising and feature extraction methods. The Butterworth filter, utilized in WIID [47], WiAu [68], and DeviceFreeAuth [56], flattens the frequency response in the passband, effectively reducing noise. Additionally, the power delay profile (PDP), as seen in WIID, characterizes the time-domain response of wireless channels, providing crucial data on signal delays and strengths. Principal component analysis (PCA), employed in NeuralWave [69] and Wii [53], reduces data dimensionality by transforming variables into linearly uncorrelated components. This decomposition helps in noise removal and reconstructing signal components. Wavelet denoising and low-pass filters, used in NeuralWave and Wii, respectively, further enhance signal clarity by filtering out noise and smoothing signals. Lastly, Gate-ID [51] utilizes bandpass filters to isolate signals within specific frequency ranges, crucial for noise reduction and signal separation in applications like communications and signal processing. This approach integrates advanced signal processing techniques to effectively extract and enhance amplitude data from CSI, enabling accurate identification of individuals based on their unique gait patterns.

Phase information in CSI is highly sensitive to subtle changes in human motion compared with amplitude characteristics. Integrating both amplitude and phase data is crucial for enhancing the robustness of HID systems based on gait analysis. However, consecutive CSI measurements may introduce varying phase offsets over time. To address this challenge, systems like NeuralWave [69,70] employ techniques to mitigate relative phase errors across subcarriers. By minimizing these offsets, they ensure more accurate and reliable identification of individuals based on their unique gait signatures. Additionally, the IndoTrack [71] study utilizes a technique known as Conjugate Phase Cancellation, which employs multiple antennas to mitigate random phase offsets by multiplying the CSI from different antennas, thereby enhancing Doppler velocity estimation.

#### 2.2.2. Gesture Analysis with CSI

Gesture analysis with CSI utilizes subtle variations in WiFi signals influenced by human gestures or movements to detect detailed aspects of human identity. CSI captures changes in the amplitude, phase, and frequency of WiFi signals, making it a powerful tool for recognizing and interpreting gestures [72]. This capability stems from the wireless channel’s ability to react to disturbances caused by body movements, as observed in various studies [73].

**Figure 5:**
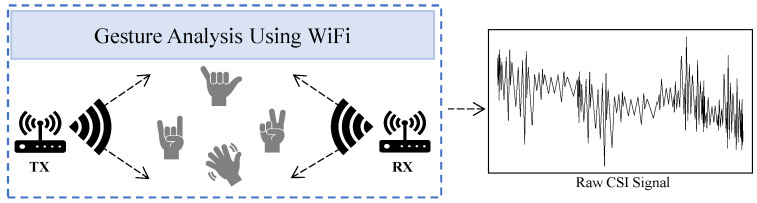
CSI raw data collection using gesture analysis via WiFi. This is the operational scenario for WiHF [76].

The WiID system [74] utilizes CSI measurements captured by WiFi receivers during user gestures. It employs PCA to remove noise from the CSI data and applies a short-time Fourier transform (STFT) to analyze the time series of these denoised CSI measurements, revealing speed patterns. The CSI data are then processed to extract frequency patterns that represent limb movement. This approach highlights that different users exhibit distinct behaviors for the same gesture. The study also finds that gesture consistency can persist over time with regular practice but may temporarily vary after extended breaks. WiGesID [75] employs a similar strategy, using PCA for noise reduction and deriving BVPs from DFSs instead of directly from CSI. Another study, WiHF [76], focuses on simultaneous gesture recognition and user identification using WiFi CSI, as shown in Figure 5. WiHF extracts motion patterns from dominant DFS components of spectrograms created through STFT.

WiDual [77] explores how gestures and user identity can be inferred from phase changes in CSI. However, obtaining dynamic phase changes in CSI faces significant challenges, including random phase offsets introduced by commercial WiFi hardware such as channel frequency offset (CFO) and sample frequency offset (SFO). To address these challenges, WiDual proposes a method to extract meaningful phase changes crucial for robust gesture and user identification. They utilize the CSI ratio method, which involves computing the quotient of CSI readings between two antennas to eliminate phase offsets. This approach transforms phase information into a two-dimensional tensor, which is further processed into an image format suitable for classification using deep learning techniques.

#### 2.2.3. Radio Biometric Analysis with CSI

Radio biometrics are defined as unique patterns in RF signal interactions influenced by individual physical attributes like height, mass, and tissue composition [78,79]. These patterns serve as distinctive identifiers analogous to fingerprints or deoxyribonucleic acid (DNA). Radio biometrics affect CSI by modulating the signal characteristics observed between transmitters and receivers, enabling the extraction of unique biometric signatures from WiFi. In recent research such as TR-HID [80] and Re-ID [81], WiFi signals’ amplitude and phase information are leveraged for precise radio biometric analysis, facilitating accurate person re-identification. Additionally, a study introduces the WiFi vision-based system 3D-ID [82], which utilizes CSI amplitude and phase information, multiple antennas, and deep learning to visualize and recognize individuals based on their body shape and walking patterns, achieving high accuracy.

#### 2.2.4. Respiration Rate Analysis with CSI

Respiration rates can uniquely identify individuals by affecting WiFi signals’ CSI. In [83], a new method analyzes respiration patterns using CSI. Unlike older approaches focused only on breathing rate estimation, this method uses both frequency and time-domain CSI data for identity matching and people counting. It includes generating multiuser breathing spectra, tracking breathing rate traces, and performing people counting and recognition. Using STFT on CSI’s amplitude and phase, it extracts breathing signals and improves the signal-to-noise ratio (SNR) with adaptive SC combining, ensuring accurate tracking and recognition. Table 1 represents the device, scene, and data used for signal collection and pre-processing in recognition based on gait, gestures, radio biometrics, and respiration rate.

### 2.3. Human Identification Methods

To identify humans, systems need to extract features such as stride length, acceleration sequences, rhythmicity, and the smoothness of gait, gesture, or radio biometric movement. In the realm of HID, researchers have demonstrated that various ML models, including both statistical or conventional ML models and neural networks, play crucial roles in extracting significant features and classifying individuals. Researchers have explored different kinds of models based on the system preferences. In this section, we will explore different HID methods, discussing both statistical ML models and advanced neural network models. We will review the research that has applied these techniques to effectively identify individuals based on their unique biometric and behavioral patterns.

#### 2.3.1. Conventional or Statistical ML Methods

Conventional or statistical ML methods use mathematical and statistical techniques to identify patterns in data, enabling predictions or classifications. In HID systems, this process starts with gathering data related to human movement, collected through wireless signals or sensors. The collected data are then carefully analyzed to extract important characteristics, followed by pre-processing steps such as data normalization, cleaning, and feature selection. This process ensures the quality and reliability of the data [85]. Classification techniques are then applied to determine human identity based on these processed data features.

**Figure 6:**
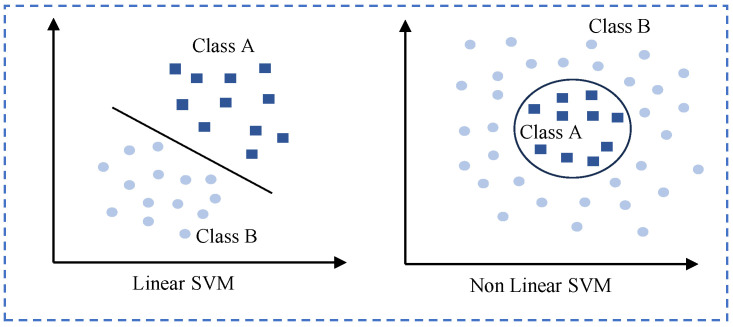
Linear data and nonlinear data in the SVM.

The SVM has been widely utilized in recent research for HID tasks. The SVM is a supervised learning model that analyzes data for classification and regression analysis. It works by finding the optimal hyperplane that best separates classes in the feature space. As illustrated in Figure 6, the SVM handles both linear and nonlinear data. This capability makes the SVM particularly effective for distinguishing between different individuals based on their extracted biometric or behavioral features. For instance, in recent studies such as Wii [53], GAITWAY [50], and Wi-IP [84], researchers have employed SVM classifiers after feature extraction to accurately identify individuals based on their gait patterns or other biometric characteristics. Wii also utilized the GMM to recognize strangers based on their behavioral patterns, and Wi-IP used linear discriminant analysis (LDA) for dimensionality reduction and feature extraction, as well as K-nearest neighbors (KNNs) for distance-based classification.

On the other hand, WiID [74] chose not to use traditional SVM methods due to their limitation in scenarios where training data are exclusively from one user (one class), while test data include samples from both the same user and others (two classes). The SVM struggles with this because it typically assumes that training and test data belong to the same class distribution. Instead, WiID implemented support vector distribution estimation (SVDE) with the radial basis function (RBF) kernel. SVDE addresses this issue by constructing separate classification models for each user’s gestures using average speed values derived from selected subseries in their samples. This method allows WiID to accurately distinguish between data from the user of interest and data from other users, without requiring extensive model retraining when users are added or removed. SVDE’s ability to handle one-class training scenarios while dealing with mixed-class test scenarios makes it suitable for WiID’s HID application. Additionally, TR-HID [80] utilized background subtraction and spatial-temporal resonance for feature extraction, while Resp-HID [83] employed multiuser breathing spectrum generation using STFT and Markov chain modeling for breathing rate tracking and people counting. Details of these models and their training specifics are provided in Table 2.

#### 2.3.2. Deep Learning Methods

**Figure 7:**
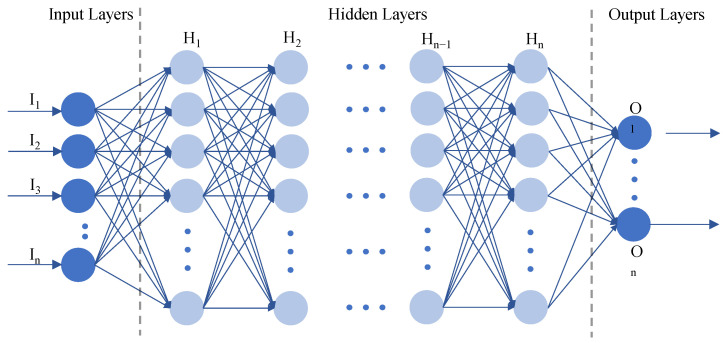
Basic neural network model.

In recent studies on WiFi CSI-based HID, there has been a notable increase in the application of neural network models such as deep neural networks (DNNs), CNNs, RNNs, and hybrid architectures. These models, inspired by the human brain’s structure where neurons and layers are interconnected (as shown in Figure 7), excel in extracting features and analyzing complex data patterns. This capability makes them effective for tasks like HID. Leveraging WiFi’s ability to capture detailed CSI, researchers are increasingly using neural networks to extract, analyze, and categorize biometric and behavioral data from these signals. This section explores the methodologies and achievements of neural network-based approaches in advancing WiFi CSI-based HID.

DNNs have become crucial for HID tasks, particularly in activity recognition and user authentication using CSI data. In [70], a DNN architecture with three stacked autoencoder layers was employed. These layers progressively extracted abstract representations from CSI data: distinguishing basic activities in the first layer, capturing activity-specific details in the second, and identifying individual users in the third. Each autoencoder layer uses nonlinear neural units to transform CSI features into abstract representations. Regularization techniques stabilize the model, and softmax functions enable hierarchical activity recognition and user authentication within each layer. Additionally, an SVM model enhances security by detecting spoofing attempts, reinforcing the system’s ability to recognize activities, authenticate users, and mitigate security risks.

CNNs are an important class of deep learning models known for their proficiency in handling structured grid data, such as images or temporal-spatial features like WiFi CSI. CNNs utilize convolutional layers that apply filters or kernels across localized regions of input data. This process allows them to extract meaningful features crucial for tasks like pattern recognition and classification. This architecture typically includes convolutional layers for feature extraction, pooling layers like max pooling for spatial dimension reduction, and fully connected layers for decision making. In recent studies on HID, CNNs have been widely employed due to their ability to automatically learn hierarchical representations from complex, high-dimensional data such as CAUTION [49], NeuralWave [69], and WiGesID [75]. CAUTION utilizes a CNN architecture with three convolutional and three max-pooling layers. This configuration not only centralizes human classification but also integrates softmax for detecting intruders. In contrast, NeuralWave employs five convolution stages, each incorporating convolutional, normalization, ReLU activation, and max-pooling layers, culminating in a fully connected layer with softmax activation for precise human recognition. In another study, LWWID [48] introduces an innovative “balloon mechanism” across its architecture. This model features four 3D convolutional layers paired with max-pooling layers. The resulting feature maps undergo concatenation, and a sigmoid function computes a crucial relation score essential for HID and gesture recognition. LWWID stands out for its unique approach in integrating 1D convolutional layers for low-level feature extraction and leveraging 3D layers for advanced feature mapping, enhancing accuracy in HID tasks [70].

Several studies have also integrated CNNs with ResNet architectures to further enhance performance in HID tasks. WiAu [68], for example, combines CNN and ResNet units within its convolutional module to achieve robust feature extraction and recognition capabilities. On the other hand, WiDual [77] adopts a dual-module approach incorporating attention-based gesture and user feature extraction. This model incorporates ResNet18 as channel attention modules, spatial attention modules for extracting features, and gradient reversal layers (GRLs) for feature fusion, ensuring nuanced results in both gesture and user recognition.

RNNs are a type of neural network that excels at recognizing patterns in sequences of data, making them especially useful for tasks involving time-based data. RNNs have loops in their architecture that allow information to be passed from one step to the next, which is essential for analyzing sequential data.

In the context of HID, ref. [56] use three kinds of RNNs: long short-term memory (LSTM), gated recurrent unit (GRU), and bidirectional gated recurrent unit (B-GRU). LSTM is chosen because it can remember long-term dependencies, which is important for tracking changes over time in location data. The GRU is used because it is efficient and still effective at handling sequences. The B-GRU unit processes data in both forward and backward directions, which helps in understanding complex dependencies in the data.

Hybrid models in the context of deep learning refer to models that incorporate features from more than two primary deep neural networks. These models are designed to leverage the strengths of different architectures, overcoming challenges that may require multiple feature extractions. Hybrid models have shown effectiveness in tasks such as gait or radio biometric feature extraction and HID, where capturing both local patterns and long-term dependencies is crucial. For instance, the fusion of CNN and LSTM networks in a hybrid model allows for the extraction of both spatial and temporal features. In CSIID [57], the basic structure encompasses three convolutional layers and one LSTM layer, enabling the extraction of spatial and temporal features crucial for comprehensive data understanding. WIID [47] employs a 1D CNN for feature extraction from WiFi CSI data, coupled with LSTM for maintaining the sequence of WiFi CSI matrices. On the other hand, GaitSense [52] utilizes a 3D CNN with 16 convolutional filters, a max-pooling layer, and a fully connected layer encoded with a softmax layer. ReID [81] employs a Siamese Neural Network Architecture, integrating CNN and LSTM in two branches. All these fine approaches, including CSIID, WIID, GaitSense, and ReID, operate on a gait-based mechanism, facilitating robust HID [70].

Furthermore, some models incorporate advanced techniques to enhance their architectures. Gate-ID [51] employs ResNet for spatial feature extraction, CNNs for data compression, dropout for regularization, and Bi-LSTM RNNs for capturing temporal patterns, demonstrating a comprehensive approach to HID. WiHF [76] uses a CNN base with a GRU for spatial feature extraction and temporal dependency capture. It also incorporates a GRL and a convolution-based RNN, enabling the extraction of detailed spatial and temporal patterns. We reviewed all the articles on HID using WiFi CSI and summarize the neural networks used and their training details in Table 3.

## 3. System Performance Evaluation

The application of the loss function and the system recognition findings based on seminal studies in CSI HID are introduced in this section.

### 3.1. Loss Function Selection

In ML models, a loss function measures the disparity between predicted outcomes and actual target values. They are used to direct model training by reducing error or loss, ensuring that the predictions made by the model closely match the desired results. We summarize the most used loss functions and their applications in Table 4.

#### 3.1.1. Categorical Cross-Entropy Loss

In classification tasks where the output belongs to one of several classes, categorical cross-entropy loss is frequently utilized. The disparity between anticipated class probabilities and actual class labels is quantified. High probabilities for the right class are rewarded, whereas high probabilities for the wrong class are penalized. This loss is appropriate for issues with classes that cannot coexist. Formulas (3)–(12) use the categorical cross-entropy loss function. In Formulas (3)–(12), consecutively, $p_{\theta }\left(y=k|x\right)$, $p_{v}$, $p_{ij}$, $b_{ym}$, $y_{ic}$, and $b_{r,k}$ defines the predicted probability. In Formula (4), we see the authors have used λΩ regularization to avoid overfitting and improve the generalization ability of the model.

#### 3.1.2. Negative Log-Likelihood Loss

In probabilistic models, negative log-likelihood loss is frequently employed and is particularly pertinent for tasks like maximum likelihood estimation in generative models. Given a probability distribution, it estimates the negative log-likelihood of the observed data. The model’s projected probabilities are brought into line with the observed data distribution by minimizing this loss. Formula (13) uses this loss function, where *L* represents the loss or the cost associated with the model’s predictions; *y* is the true target or label; and *p* is the predicted probability.

#### 3.1.3. Mean Squared Error (MSE) and Mean Absolute Error (MAE)

MSE and MAE are another type of loss function for regression tasks and are preferred when you want to measure the average absolute difference between predicted and actual values. MSE calculates the average of the squared differences between predicted and actual values. MSE penalizes larger errors more severely, making it suitable for tasks where precise numerical prediction is important. Unlike MSE, which squares errors, MAE takes the absolute value of errors. It is less sensitive to outliers and can provide a more robust measure of error when dealing with data containing significant outliers. CAUTION [49] uses these loss functions to compare with the categorical cross-entropy loss function, where cross-entropy performs better than these two functions.

### 3.2. Performance Analysis of HID

This section analyzes the WiFi CSI-based system performances in HID papers, as shown in Table 5. One notable system, WiID [47], demonstrates promising results. When augmented with data, WiID achieves a remarkable 98% accuracy with just two humans, and maintains a high 92% accuracy even with eight humans. Without data augmentation, it still manages to attain a respectable 98% accuracy with two humans, albeit slightly lower at 85% with eight humans. This suggests that WiID is particularly effective in scenarios with limited human presence and can maintain good performance, even as the number of humans increases.

Another system, NeuralWave [69], reports an accuracy of 87.76% under unspecified conditions. While it does not reach the same level of accuracy as WiID, it still presents a viable option for HID tasks. WiAu [68] achieves approximately 98% accuracy, similar to WiID, but with a focus on 12 humans. LW-WiID [48] exhibits remarkable performance with 100% accuracy when the number of humans falls within the range of 10 to 20, and an impressive 99.7% accuracy with 50 humans. These findings indicate that LW-WiID excels in scenarios with a larger number of humans, making it suitable for various applications, especially those requiring crowd monitoring or large-scale user authentication.

In addition to these systems, there are several others like GaitSense [52], CAUTION [49], GateID [51], CSIID [57], and Device-FreeUserAuth [56,70], each with its own accuracy rates and conditions. These systems cater to different use cases and scenarios, ranging from office environments to apartments, and exhibit varying levels of performance. Overall, these systems showcase the advancements in HID technology, offering a range of solutions for diverse applications, from security to user authentication and crowd monitoring.

## 4. Discussion

In this section, we will explore the unique and additional applications integrated into various HID systems. Some innovative systems extend their functionality to include intruder or stranger detection, enhancing security by identifying unauthorized presence. Others utilize alternative data sources, such as BVPs instead of directly using CSI, to achieve more robust and accurate identification. These diverse approaches highlight the versatility and potential of HID systems in addressing complex real-world challenges.

### 4.1. User Authentication and Intrusion Detection

Many systems often overlook the presence of unknown intruders within an environment, a critical factor that can significantly impact accuracy rates. However, some authors have recognized the importance of addressing this challenge and have endeavored to develop systems capable of detecting such individuals. In Wii [53], the model is trained using data from both authenticated individuals and individuals categorized as strangers. This means the system does not detect truly unknown people. The accuracy tends to decrease as the number of strangers in the training set increases because the features of strangers become so generally representative that they overshadow the features of authenticated people. Therefore, the number of strangers in the training set should be limited. To address this issue, other researchers have developed solutions that do not require data from strangers at all. Instead, they use threshold-based or algorithmic approaches to detect intruders. These methods enable the system to identify unauthorized individuals without needing a pre-collected dataset of stranger profiles. This approach enhances the robustness and scalability of HID systems. The following section will discuss three existing research studies on HID, categorizing them into two main approaches: threshold setting-based and algorithm-based techniques.

#### 4.1.1. Threshold Setting-Based Technique

The threshold setting-based approach involves detecting intruders and threats by establishing a specific threshold. This threshold can be determined empirically, using domain knowledge or extensive experiments. Alternatively, it can be optimized through an iterative process, selecting an optimal threshold value based on predefined criteria. This iterative process is often referred to as metric-driven threshold optimization.

A DNN is employed to extract representative features of human activity details for user authentication in [70]. Additionally, a one-class SVM model is utilized for HID. In this HID approach, the threshold value, η, is determined empirically. The process involves constructing a one-class SVM model for each authorized user using features derived from high-level abstractions obtained from three-layer DNN networks. For a given testing sample abstraction, *Z*, a class score, Su(Z), is as follows.

(18) $$Su\left(Z\right)=\sum_{i}k\left(Z_{u,i},Z\right)+b_{u},$$

where Su(Z) is computed for each user, *u*, by comparing the resemblance between the test sample’s DNN abstractions, *Z*, and the support vector *i*th of the user, *u*, using a Gaussian kernel function, k(). A higher class score, $S_{u}$, indicates less distance between the testing sample and the user’s support vectors.

An empirically set threshold, η, is then used to classify testing samples as potential intruders. If the class scores, $S_{u}$, for a testing sample are lower than the threshold, η, for all legitimate user profiles, the sample is classified as a potential intruder. The threshold, η, is determined based on the application requirements and the desired trade-off between false positives (incorrectly identifying legitimate users as intruders) and false negatives (failing to detect actual intruders). It may be set through experimentation or domain knowledge to achieve the desired level of security and performance.

In [70], the authors experimented with intruder detection in an office area, achieving an accuracy of 89.7% for both intruder and authorized user detection through a balanced trade-off. Empirically set thresholds offer advantages such as reduced computational overhead, adaptability to new environments, and flexibility to accommodate new data. However, determining optimal threshold values may require manual intervention or expertise, which can be time-consuming and subjective.

As metric-driven thresholds are determined through an optimization process, they ensure that the threshold is set at an optimal value for distinguishing between known and unknown samples. In CAUTION [49], the system receives CSI samples that are processed using a CNN network by a feature extractor, generating corresponding low-dimensional representations on the feature plane. The model then computes distances and selects the first two nearest central points, $C_{1st}$ and $C_{2nd}$. With these distances, CAUTION calculates the Euclidean distance ratio, *R*.

(19) $$R=\frac{d(F_{\theta }\left(x_{n}\right),C_{1st})}{d(F_{\theta }\left(x_{n}\right),C_{2nd})},$$

here, if *R* is less than or equal to the threshold, *T*, $x_{n}$ is classified to the class of $C_{1st}$; otherwise, it is classified as an intruder.

To accurately detect intruders, optimizing the threshold, *T*, is crucial. The training data {X,Y} from *k* users are divided into two groups: known users’ data and unknown users’ data. Known users’ data are further divided into training ($X,Y_{t}$) and validation ($X,Y_{v}$) sets. The system is trained on user identification using the training set $X,Y_{t}$, while the validation set, $X,Y_{v}$, is used to evaluate the system’s performance and optimize the intruder threshold. A range of threshold values, *T*, is selected from 0 to 1. CAUTION tries out *I* different values of the threshold, *T*, within the selected range, evenly distributed. For each value of *T*, CAUTION calculates the distance ratio, *R*, for each CSI sample in the validation set and compares it with the threshold. The threshold value that provides the best performance in distinguishing between unknown and known samples is selected. Once the initial threshold value is identified, the process is refined by selecting another range of threshold values around the best-performing threshold. This refinement is repeated $N_{iter}$ times until the best threshold value is determined.

With this threshold optimization approach, CAUTION achieves an accuracy rate of approximately 98% to 88% across different user group sizes from 6 to 15. One advantage of metric-driven thresholds is their ability to adapt to changes in the dataset or environment by dynamically adjusting the optimality based on performance metrics. However, there is a risk of overfitting the training data if the optimization process is not carefully controlled.

#### 4.1.2. Algorithm-Based Technique

Threshold setting-based HID techniques are sensitive to environmental changes. Therefore, GaitSense [52] employs an algorithm-based approach that is not affected by such changes. In GaitSense, CSI data are processed through a CNN network to extract a 128-dimensional feature vector from the LSTM output. The system then completes the intruder detection process in three steps.

First, during training with only authorized users, GaitSense calculates the mean average neighbor distance for each legitimate class to its *K* nearest neighbors, setting this average value as the class density in the feature space. Second, during testing with authorized and unauthorized users, GaitSense identifies the *K* nearest neighbors of the test samples and determines the most common class among these neighbors, considering it as the potential class for the test sample. Lastly, for each test sample, GaitSense calculates the mean distance from the sample to its *K* nearest neighbors within the potential class. The model then compares this mean distance to a threshold parameter multiplied by the density of the potential class (calculated in the first step). If the mean distance exceeds the weighted class density, the test sample is classified as an intruder; otherwise, it is considered an authorized user.

GaitSense’s data extraction process is based on body velocity rather than the Doppler effect, making the algorithm-based approach inherently resilient to environmental variations such as signal attenuation and channel fading. This ensures consistent performance regardless of changing conditions. However, the feature extraction process in GaitSense is complex and resource-intensive. Table 6 presents a comparison of all the intruder detection techniques discussed.

### 4.2. Utilization of BVPs

BVPs are a feature derived from WiFi CSI that characterize the velocity components of human movements within a specific coordinate system attached to the body. Unlike traditional CSI, which captures the propagation characteristics of WiFi signals and their interaction with the environment, BVPs focus on extracting motion-related information directly associated with the human body during activities such as gestures or gait. Some research has increasingly favored BVPs over traditional CSI for applications involving human motion recognition. This shift arises from the limitations of CSI in accurately capturing dynamic human movements. CSI measurements are affected by environmental factors, such as multipath interference and signal attenuation, which can distort the fidelity of motion-related data. In contrast, BVPs provide a more direct representation of human movement dynamics, making them a preferred choice in scenarios where precise motion tracking is critical.

The adoption of BVPs addresses several challenges associated with using CSI for motion recognition. CSI is receptive to environmental changes that can alter signal propagation characteristics, leading to inconsistencies in motion detection. BVPs, by focusing on body-centric velocity profiles, mitigate these environmental impacts, ensuring more reliable motion analysis. Additionally, BVPs simplify the extraction process compared with CSI, which requires complex algorithms for accurate interpretation, making them more suitable for real-time applications where computational efficiency is crucial.

WiGesID [75] leverages the advantages of BVPs to enhance the accuracy and robustness of gesture recognition systems. By utilizing BVPs, WiGesID can effectively capture the nuanced motion dynamics of various gestures, which are often challenging to discern using traditional CSI due to environmental noise and interference. WiGesID implements a framework that collects CSI data and processes them to generate BVP features, which then serve as the basis for recognizing different human gestures. This approach allows WiGesID to maintain high recognition accuracy even in complex and variable environments, as the body-coordinate system inherently filters out irrelevant environmental factors. Moreover, the use of BVPs in WiGesID enables the system to operate with lower computational overhead, making real-time gesture recognition feasible. The success of WiGesID in employing BVPs underscores the feature’s potential to deliver precise and efficient motion recognition solutions.

Another notable application of BVPs is in GaitSense [52], a WiFi-based HID framework designed to be robust to various walking manners and environmental changes. GaitSense employs BVPs to effectively model the kinetic characteristics of human gait, overcoming the limitations of traditional CSI-based approaches. GaitSense first pre-processes CSI measurements to remove phase noise and then performs motion tracking and time-frequency analysis to generate the BVP. The BVP provides a comprehensive profile of body movements by capturing the power distributions over velocity components within a coordinate system centered on the body. This makes it possible to accurately track gait patterns regardless of the person’s location or orientation relative to the WiFi devices.

To formulate the BVP, GaitSense uses the following equations. The BVP, denoted as *G*, is derived from the DFS observed in the CSI data. For a single reflection path, the relationship between the velocity components, $v_{x}$ and $v_{y}$, and the Doppler frequency, $f_{D}$, is given as follows.

(20) $$f_{D}=a_{x}v_{x}+a_{y}v_{y},$$

where $a_{x}$ and $a_{y}$ are projection coefficients determined by the locations of the transmitter, receiver, and target. The BVP is then constructed by minimizing the Earth Mover’s Distance (EMD) between the observed DFS and the reconstructed DFS from the BVP. This optimization problem is expressed as follows.

(21) $$\mathrm{GBVP}=\min_{G}\sum_{i=1}^{L}\left|\mathrm{EMD}\left(D^{\left(i\right)}\left(G\right),{\mathrm{DFS}}^{\left(i\right)}\right)\right|+\eta {∥G∥}_{0},$$

where *L* is the number of WiFi links, ${\mathrm{DFS}}^{\left(i\right)}$ is the observed DFS on the *i*-th link, and η is a sparsity coefficient. By using BVPs, GaitSense can accurately identify individuals based on their gait patterns, achieving high identification accuracy with significantly reduced training data compared with traditional methods. The system is designed to be agile and adaptive, capable of quickly integrating new users with minimal data while maintaining robust performance. The success of GaitSense in utilizing BVPs demonstrates the feature’s effectiveness in providing reliable and efficient gait-based HID.

## 5. Future Research Trends

This study provides a detailed and organized review of the expanding field of WiFi CSI-based person identification methods. It performs a thorough analysis of the classification and functionality of numerous WiFi-based person identification apps. Although this sector has made great advancements, it is important to recognize that there are still many obstacles to overcome, which highlights the demand for additional study and innovation in the field of HID utilizing WiFi CSI data. Some proposed future research trends can be summarized as follows.

### 5.1. Exploring Transfer Learning

Transfer learning holds significant promise for overcoming challenges faced in WiFi-based HID systems. By leveraging knowledge from one domain and applying it to another, transfer learning can help mitigate issues related to limited data, adaptability to new environments, and cross-domain applications. The key areas where transfer learning can drive progress are as follows.

The current datasets for WiFi-based HID systems, for example, Widar3.0 [64] and WiAR [65], are limited in size, type, and extent. These datasets encounter some challenges due to the multipath effect and sometimes due to the different environments where they are applied. This lack of adequate and comprehensive datasets hinders effective model training and generalization. However, transfer learning is an efficient approach, as it enables the models to use the knowledge from relevant but different datasets in the same domain. It reduces the dependency on large data collection for effective training. Such characteristics are essential to fine-tuning HID systems for specific tasks or environments. For example, in [86], the authors used transfer learning to employ modified pre-trained architectures like ResNet18 in the Wi-AR [86] system. In this way, one can achieve better performance in HAR systems, for example, in a situation with a small number of training data, by preventing overfitting and computational burdens in small sample conditions.

It is commonly known that WiFi signals are not constant in different physical scenarios due to shadows, attenuation effects, or signal interference. A model that is usually trained for a particular environment tends to lose its accuracy in a different environment. Transfer learning may be more effective for the reason that pre-trained models from related domains can be modified to fit the new environment instead of training the model from the very beginning [87]. This approach allows the system to automatically optimize for changing environmental factors, thereby enhancing the reliability of HID applications.

Transfer learning enables the use of models developed for one problem in the human identification domain in another. This includes, but is not limited to, gait recognition, gesture recognition, or even different sensing technologies, such as transitioning from WiFi to radar identification. This ability to leverage features and representations learned in each domain allows a WiFi-based HID system to be applied to other tasks without starting over with fresh training from scratch. Furthermore, this cross-domain transfer can enhance the applicability and usefulness of these systems in multitasking and multipurpose configurations, which reflect real-world scenarios.

### 5.2. Handling Dynamic Environments

Dynamic environments pose significant challenges for WiFi-based human identification (HID) systems due to factors such as walking individuals, moving objects like furniture, and other dynamics within a location. These movements create interferences, compromising the accuracy of the signals and hampering any practical use of the systems. In these circumstances, further studies should focus on more effectively separating human movement from the motion of other objects. The BVP technology is particularly effective in monitoring an individual’s movement in real time, as it captures very small velocities, making it easier to distinguish between actions performed by a person and movements caused by the environment [88]. Moreover, all the information captured during the application of feature-enhanced walking pattern recognition can be thoroughly processed. Irrelevant information above the baseline signal can be filtered out using sensor technology, promoting the recognition of walking and other gestures. Additionally, adaptive approaches, likely combining WiFi sensing and machine learning techniques, can effectively address dynamic situations. This combination can enhance the applicability of WiFi-based HID systems, making them more effective and reliable.

### 5.3. Enabling Multiuser Identification

Moving from theory to practical applications, WiFi-based HID systems cannot avoid multiuser situations, which increases the level of difficulty. Generally, current systems are typically designed for single-user operation, limiting their usability in cramped or communal settings such as homes, offices, or public spaces. Multiuser identification encompasses numerous techniques to identify individuals behind multiple coexisting signals in the same area, including signal separation and clustering to track the movements of different individuals. To achieve this, there is a need to design efficient spatial resolution in future work, perhaps through advanced antenna arrays or more extensive studies in MIMO technology. This will enhance spatial resolving power, making it easier to distinguish users occupying the same space. Furthermore, multilingual architectures can be incorporated into deep learning frameworks to create effective learning processes in multiuser environments [89]. Such improvements would facilitate the prompt recognition of multiple individuals simultaneously, paving the way for WiFi HID systems that are more practical and easier to implement.

## 6. Conclusions

This study reviews recent advancements in human identification using WiFi and ML. It examines the identification process with CSI from activities like gait, gestures, radio biometrics, and respiration rates, emphasizing the pre-processing methods required to extract features for ML models. The review covers various ML models, including traditional statistical methods and deep learning approaches, highlighting their applications in human identification. The paper also discusses the current state of WiFi-based sensing methods, identifying key challenges and future research trends. The conclusion summarizes the findings, emphasizing the need to address identified challenges to improve WiFi-based human identification. It also suggests future research directions to advance the field, guiding researchers to extend their work and overcome existing limitations.

## Figures and Tables

**Table 1:** Device, scene, and data for signal collection.

System	Device	Signal and Preprocess	Experiment	Amount of Data
WIID [47]	One transmitter and one receiver; Intel 5300 NIC; 5.32 GHz frequency with 20 MHz	Amplitude; PDP, Butterworth filter for denoising; sliding window, time warping for data augmentation	8 subjects (5 males, 3 females); in classroom; on subjects’ behaviors (e.g., using a smartphone, running, walking, jumping, etc.)	100 datasets each class; up to 9 classes 70%: training; 30%: testing
WiAU [68]	WiFi router as transmitter and laptop as receiver; TP Link TL WR886N; Intel 5300 NIC	Amplitude; Butterworth low-pass filter for denoising; sliding window for data transformation	12 to 16 volunteers; two scenes: office and three corridors; walking and 16 activities	60%: training; 20%: validation; 20%: testing
Gate-ID [51]	WiFi router as transmitter and HP 8530p laptop as receiver; Netgear R7000; Intel 5300 NIC; 5.19 GHz frequency with 40 MHz	Amplitude; bandpass filter	20 college students; indoor smart home or small office; walking	200 walking observation; 60%: training; 20%: validation; 20%: test
GaitSense [52]	One transmitter and six receivers; Intel 5300 NIC; 5.825 GHz	Amplitude and phase	11 volunteers (4 females, 7 males); an empty discussion room with desks and chairs and a wide hall beside stairways; walking	11 subjects and overall 4600 traces; 50–400 gait samples from each user
CSIID [57]	WiFi router as transmitter and notebook as receiver; TPLINK AC1750 router; Intel 5300 NIC; 5 GHz frequency band	Amplitude; sliding window processing method to select sample	6 participants (3 males, 3 females); indoor environment; 5 s walking time with 100 times walking cycles	70%: training; 30%: testing
LW-WiiD [48]	WiFi router as transmitter and receiver; TP-Link router; Intel 5300 NIC; 5 GHz	Amplitude; frequency energy graph (FEG)	10 volunteers (7 males, 3 females); big classroom, laboratory, meeting room, psychological counseling, and an exhibition room; walking	80%: training; 20%: testing
CAUTION [49]	WiFi router as transmitter and receiver; TP-Link N750 router; 5 GHz with 40 MHz	Amplitude	20 volunteers (12 males, 8 females); 15 volunteers grouped as legal users; rest as illegal intruders; lab and cubic office; walking	2 to 15 users; 20, 40, and 100 CSI samples from each user
NeuralWave [69]	Gateworks GW5400 SBC as WiFi transmitter and laptop as receiver; Intel 5300 WiFi card; 5 GHz with 40 MHz	Amplitude and phase; missing data imputation, phase calibration, wavelet denoising, PCA for dimension reduction	24 volunteers; indoor laboratory; walking	40 CSI samples; 70%: training; 30%: validation;
Shi et al. [70]	Dell E6430 laptop as transmitter and Lenovo laptop as receiver; Intel 5300 NICs	Amplitude and phase; phase calibration, band-pass filtering to remove noise, subcarrier selection to acquire valid CSI measurements	11 volunteers in university office; 5 volunteers in apartment; walking activities and stationary activities	50%: training; 50%: testing
Jayasundara et al. [56]	WiFi router as transmitter and receiver; TP-Link N750 router; 5 GHz with 40 MHz	Amplitude; sparsity reduction operation, Butterworth filtering to remove the noise, concatenate the filtered CSI measurements	13 participants; lecture room; sit, jump, fall, run, NoAc, walk1, walk2, walk3, walk4	–
Wii [53]	TP-Link WiFi router as transmitter and laptop as receiver; Intel 5300 NIC	Time and frequency domain; PCA and low- pass filter; CWT	Eight healthy volunteers; meeting room (with a meeting table and chairs); walking	8 volunteers; 150 steps each; approx. 10 to 50 steps training set
GAITWAY [50]	One laptop as transmitter and one laptop as receiver; Intel 5300 NIC; 5.8 GHz frequency with 40 MHz	Amplitude and phase	11 human subjects, 5 female, and 6 male; a typical office space; walking; read news, play mobile games, or talk on the phone while walking	Gait instances: 5283; ≈70%: training; ≈30%: testing
Wi-IP [84]	One TP-Link router as transmitter, one ThinkPad laptop with three antennas as receiver; 2.4 GHz frequency with 20 MHz	Amplitude; Savitzky–Golay filter for denoising, normalization, filtering, and smoothing	6 volunteers, 3 males, and 3 females; indoor corridor; walking (3.5 m per walk)	90%: training; 10%: testing
WiGesID [75]	One transmitter and three receivers; off-the-shelf mini-desktops equipped with Intel 5300 NIC; 5.825 GHz	Amplitude; time-frequency transformation, low-pass filter and PCA, derives BVPs from DFS	16 volunteers, 12 males and 4 females; classroom, hall and office; with almost 16 different gestures	≈70%: training; ≈30%: testing
WiHF [76]	One transmitter and three receivers; off-the-shelf mini-desktops equipped with Intel 5300 NIC; 5.825 GHz	Amplitude; band-pass filtering, PCA to reduce dimensionality, STFT to obtain DFS	16 volunteers, 12 males and 4 females; classroom, hall and office; 6 common hand gestures and 10 other gestures	80%: training; 20%: testing
WiDual [77]	One transmitter and three receivers; off-the-shelf mini-desktops equipped with Intel 5300 NIC; 5.825 GHz	Phase; CSI image conversion	15 users; classroom, hall and office; with 20 gestures	80%: training; 20%: testing
WiID [74]	One transmitter and one receiver; Intel 5300 WiFi NICs with three omnidirectional antennas, connected to a NETGEAR R6700 access point; 2.4 GHz	CSI frequency component PCA for denoising	15 volunteers (9 males and 6 females); lab, office, living room, and bedroom	80%: training (1575 samples); 20%: testing (175 samples)
TR-HID [80]	One 3-antenna transmitter and one 3-antenna receiver; 5.845 GHz with 40 MHz	Amplitude and phase	11 users; 10 floors of commercial office building with elevator; walking and other activities	Training: 50 CSI testing: 500 CSI samples per class
Re-ID [81]	802.11n commercial router as transmitter and desktop pc as receiver; Intel 5300 NIC; 2.4 GHz with 20 MHz	Amplitude and phase	35 people (15 women and 20 men); indoor (three scenarios: conference room, office, hallway); standing in between transmitter and receiver; total 525 transmissions are taken	≈80%: training; ≈20%: testing; 20 individuals with total 300 transmission
Resp-HID [83]	One as transmitter and one as receiver; WiFi device with three omnidirectional antennas; 5.765 GHz with 40 MHz	Amplitude and phase	12 participants; campus, lab, and a car over two months	–

**Table 2:** Statistical or conventional ML model.

System	Activity Type	Model Implementation	Training Details
Wii [74]	Gait based	Feature extraction, classification (stranger recognition and identifying individuals); GMM to recognize stranger, SVM to identify individuals	Radial basis function kernel; holdout cross-validation
GaitWay [50]	Gait based	Extracting gait features, recognizing gait using SVM	Radial basis function kernel; grid search with 10-fold cross-validation to select optimal values of parameters “g” (gamma) and “c” (regularization parameters); feature scaling [0,1] range for classification
Wi-IP [84]	Gait based	SVM-based model with RBF kernel for classification, by using gait energy and various time-domain and frequency-domain features	10-fold cross-validation was used to verify the model
WiID [74]	Gesture based	Extracting gesture features using time series segmentation, gesture and user classification using support vector distribution estimation (SVDE)	Radial basis function kernel (RBF); grid search with 10-fold cross-validation to select optimal values of parameters γ (RBF kernel) and ν (SVDE); feature scaling [0,1] range for classification
TR-HID [80]	Radio biometric	Background subtraction; feature extraction (spatial-temporal resonance); calculating time-reversal spatial-temporal resonance; dimension reduction	Background subtraction factor α = 0.5; threshold, μ = 0.9 for human identification in performance metrics
Resp-HID [83]	Respiration rate	Multiuser breathing spectrum generation using short-term Fourier transform (STFT); breathing rate trace tracking by using Markov chain model and iterative detection and projection; people counting and recognition by using hypothesis testing	–

**Table 3:** Neural network and training details.

Model	System	Activity Type	Neural Network Implementation	Training Details
CNN	LW-WiiD [48]	Gait based	Balloon mechanism that works as feature extractor, feature processing (consists of 1 × 1 conv and 3 × 3 conv)	Higher learning rate because of batch normalization
	CAUTION [49]	Gait based	Feature extraction: three convolutional layers (3 × 3 kernel); three max-pooling layers to convert large dimensional CSI samples into low dimension	Few shot learning
	WiAu [68]	Gait based	1 CNN layer and 15 ResNet layers; kernel of CNN is 7 × 7, ResNet is 1 × 1 and 3 × 3, respectively	Transfer learning
	NeuralWave [69]	Gait based	First layer input layer is the size of 354 × 1; remaining 22 layers are organized into six stages; five convolutional stages for feature learning and extraction; following one convolution layer, one batch normalization layer, one ReLU activation layer, and one max-pooling layer; one fully connected layer, and one output layer	Adam optimizer; learning rate: 0.001; decay rate: 0.0001; epochs 20; mini-batch size: 32
	WiGesID [75]	Gesture based	Dual-task conv3D model; consists of four parts: an input layer (20 × 20 × 34), a conv3D encoder (four 3D convolutional layers and three pooling layers), a feature concatenation layer (for semantic and identity recognition, respectively), and a relational comparison layer	Learning rate 0.001; batch size 19
	WiDual [77]	Gesture based	ResNet18 is used to apply channel and spatial attention modules as feature extractor of gesture and user; after feature extraction both features from users and gestures are concatenated using a GRL	Learning rate 0.001; batch size 20
DNN	Shi et al. [70]	Gait based	A three-layer stacked autoencoder, SVM model to detect spoofer	–
RNN	Jayasundara et al. [56]	Gait based	Three different types of recurrent layers LSTM, GRU, and B-GRU	Adam optimizer; learning rate 0.001; for user authentication dropout rate 0.3 and tracking dropout rate 0.2; epochs 60
Hybrid model	CSIID [57]	Gait based	CNN+LSTM; the basic structure includes the convolution layers and LSTM layers; three convolutional layers and one LSTM layer	Adam optimizer; learning rate 0.0001
	WIID [47]	Gait based	CNN+LSTM 1D CNN: feature extraction from input WiFi CSI data, convolution filters (100 × 120 and 100 × 10), max pooling and 0.001 s of temporal resolution; LSTM: maintain, process, and ensure sequence of WiFi CSI matrix	Central points for each user
	GaitSense [52]	Gait based	CNN+LSTM 3D CNN, 16 convolutional filters (size 5 × 5 × 5), output (size 16 × 16 × 26); max-pooling layer (size 8 × 8 × 26) and a fully connected layer LSTM: encoded with softmax layer	Transfer learning
	Re-ID [80]	Radio biometric	Siamese neural network architecture CNN+LSTM two branches, each with parallel sub-networks (CNN and LSTM); CNN analyzes amplitude heatmaps, the LSTM processes phase sequences; then both CSI measurements are concatenated	Stochastic gradient descent (SGD) for optimization; each model is trained for 200 epochs; learning rate 0.1; weight decay 5 × 10^−4^; Nesterov momentum 0.9
	Gate-ID [51]	Gait based	ResNet+RNN+CNN+Bi-LSTM ResNets for spatial feature extraction, CNNs for further compression, dropout for regularization, Bi-LSTM RNNs for capturing temporal patterns	Learning rate 0.0008; dropout rate from 0.1 to 0.7
	WiHF [76]	Gesture based	CNN+RNN CNN base GRU is adopted; extracting spatial features using a CNN and temporal dependencies using a GRU; splitting and splicing using GRL	Early stopping using the patience epochs 30

**Table 4:** Loss function selection.

Loss Function	Application	Formula	
Categorical cross-entropy loss	CAUTION [49]	$L\left(\theta \right)=-\log p_{\theta }\left(y=k|x\right)$	(3)
	LW-Wiid [48]	$Loss=-\sum_{v=1}^{H}q_{v}\log\left(p_{v}\right)$	(4)
	WIID [47]	$L=-\sum_{i=1}^{N}\sum_{j=1}^{C}y_{ij}\log\left(p_{ij}\right)$	(5)
	WiAu [68]	$Loss1=-\frac{1}{d}\sum_{m=1}^{d}y_{m}\log\left(b_{ym}\right)+\lambda \Omega$	(6)
	NeuralWave [69]	$L\left(t,y\right)=-\sum_{i=1}^{N}\sum_{c\leq C}t_{ic}\log\left(y_{ic}\right)$	(7)
	Jayasundara et al. [56]	$L_{\mathrm{activity}}=-\frac{1}{R}\sum_{r=1}^{R}\sum_{k=1}^{K}a_{r}\log\left(b_{r,k}\right)$	(8)
	WiDual [77]	$L\left(\theta ,D\right)=\frac{1}{2}\sum_{t=1}^{n}\sum_{i}\log\left(1+\exp\left(-{\hat{y}}_{i}^{t}\cdot y_{i}^{t}\right)\right)$	(9)
	WiGesID [75]	$L_{\mathrm{cel}}\left(X,Y,W\right)=\frac{1}{2}\sum_{t=1}^{2}\sum_{i}\log\left(1+\exp\left(-{\hat{y}}_{i}^{t}\cdot y_{t}^{i}\right)\right)$	(10)
	WiHF [76]	$L=-\sum_{i=1}^{N}\sum_{j=1}^{C}y_{ij}\log\left(p_{ij}\right)$	(11)
	Re-ID [80]	$L\left(D\right)=-\sum_{D_{d}}y_{d}\log\left(\frac{\exp(d)}{\sum_{D_{d}^{\prime }}\exp\left(d^{\prime }\right)}\right)$	(12)
Negative log-likelihood loss	Gait-ID [51]	L=−[ylog(p)+(1−y)log(1−p)]	(13)
	Resp-HID [83]	$C\left(g\right)=-\log P\left(g\left(1\right)\right)-\sum_{i=2}^{I}\log P\left(g\left(i-1\right),g\left(i\right)\right)$	(14)
Error cost function	Shi et al. [70]	$\mathrm{ERR}\left(X,\hat{X}\right)=\frac{1}{N}\sum_{n=1}^{N}{\left(X_{n}-{\hat{X}}_{n}\right)}^{2}+\lambda \cdot \Omega_{\mathrm{weights}}+\beta \cdot \Omega_{\mathrm{spar}\;\mathrm{sity}}$	(15)
Mean squared error	CAUTION [49]	$MSE=\frac{1}{N}\sum_{i=1}^{N}{\left(y_{i}-{\hat{y}}_{i}\right)}^{2}$	(16)
Mean absolute error	CAUTION [49]	$MAE=\frac{1}{N}\sum_{i=1}^{N}\left|y_{i}-{\hat{y}}_{i}\right|$	(17)

**Table 5:** System performance.

Model	User Identification Performance
WIID [47]	With data augmentation: N = 2, 98%; N = 8, 92%; without data augmentation: N = 2, 98%; N = 8, 85%
NeuralWave [69]	87.76 ± 2.14%
WiAu [68]	N = 12, ∼98%
LW-WiID [48]	N= 10–20, 100%; N = 50, 99.7%
GaitSense [52]	GBVP samples: ∼50; N = 2, 99%+; N = 5, 93.2%; N = 11, 76%
CAUTION [49]	CSI samples: 100; in lab: N = 2, 99.67%; N = 8, 95.18%; N = 15, 87.89%; in cubic: N = 2, 99.63%; N = 8, 93.91%; N = 15, 88.00%
GateID [51]	N = 6 to 20; 90.7% to 75.7%
CSIID [57]	N = 2 to 6; 97.4% to 94.8%
Jayasundara et al. [56]	97.41%
Shi et al. [70]	In office: N = 11, 92%; in apartment: N = 4, 90%
WiID [74]	Case 1 (height): N = 2, 93%; N = 3, 90%; N = 5, 94%; case 2 (weight): N= 2, 92%; N = 3, 89%; N = 5, 93%; case 3 (light winter clothing): N = 2, 91%; N = 3, 90%; N = 5, 92%; case 4 (heavy winter clothing): N = 2, 79%; N = 3, 81%; N = 5, 81%
GAITWAY [50]	Verification rate: 90.4%; recognition rate: N = 5; 81.2% and N = 11; 69.8%
Wi-IP [84]	N = 2, 100%; N = 3, 97%; N = 4, 90%; N = 5, 85%; N = 6, 80%
Wii [53]	Stranger recognition: N= 1; 91% and N= 5; 80%; identification accuracy: N = 8, 90.9% and N = 2; 98.7%
WiHF [76]	N = 6; gesture recognition: 97.65%; user identification: 96.74%; N = 9; gesture recognition: 93.11%; user identification: 95.33%
WiGesID [75]	New gesture categories: 94.1%; new user categories: 93.2%
WiDual [77]	Gesture recognition: 98.58%; user identification: 98.73%; in cross-domain situations for each recognition task: over 90%
TR-HID [80]	Human identification: 98.78%
Resp-HID [83]	N = 4; 85.78%

**Table 6:** Comparison table for human intruder detection techniques.

Reference	System Model	Technique	Classification	Advantage	Disadvantage
Shi et al. [70]	DNN	Empirically set threshold η[−1,1]	SVM	Less computational overhead; flexible to diverse dataset; adaptability to new environment	Time consuming; requires manual intervention or expertise to find optimal threshold values
CAUTION [49]	CNN	Metric-driven threshold 0<T<1	Euclidean distance	Finds optimal threshold value dynamically regardless of dataset and environment changes	Risk of overfitting to the training data if not controlled
GaitSense [52]	CNN + LSTM	Algorithm based	KNN	Resilient to environmental changes; unaffected by signal attenuation or fading	Complexity and resource-intensive
